# Artificial intelligence-driven triage in emergency departments: a comparative study of machine learning approaches

**DOI:** 10.1590/1806-9282.20260287

**Published:** 2026-10-05

**Authors:** Ayşe Şule Akan, Sefa Kucuk, Şule Yakar, Mahmut Tutam, Hatice Esen, İbrahim Toker

**Affiliations:** 1 Erzurum City Hospital, Department of Emergency Medicine – Erzurum, Türkiye.; 2 Erzurum Technical University, College of Engineering and Architecture, Department of Electrical and Electronics Engineering – Erzurum, Türkiye.; 3 Kayseri City Hospital, Department of Emergency Medicine – Kayseri, Türkiye.; 4 Erzurum Technical University, College of Engineering and Architecture, Department of Industrial Engineering – Erzurum, Türkiye.

**Keywords:** Emergency departments, Machine learning, Triage

## Abstract

**OBJECTIVE:**

In emergency departments, accurate and timely triage plays a critical role in prioritizing patient care. Traditional triage often suffers from subjectivity and inconsistency, especially under time pressure and overcrowded conditions. The aim of this study was to evaluate the predictive potential of multiple supervised machine learning models for three-class emergency department triage classification and compare their performance using routinely available triage variables.

**METHODS:**

We conducted a comparative analysis of eight supervised machine learning models using a retrospective dataset comprising 510 patient records from the emergency department triage. The dataset included demographic features, vital signs, and chief complaints. A 10-fold cross-validation strategy was employed to evaluate performance in a three-class triage outcome scenario based on urgency levels using accuracy, precision, recall, and F1-score.

**RESULTS:**

Among the evaluated models, logistic regression, random forest, support vector machine, and majority voting achieved the highest predictive performance, with overall accuracies exceeding 89%. The confusion matrix analysis revealed that low-urgency cases were consistently classified with the highest accuracy across all models, while misclassifications occurred primarily between high-urgency and medium-urgency cases.

**CONCLUSION:**

The results show that machine learning models appear well-suited to support the emergency departments triage decision-making process. Integrating such models into emergency departments workflows can improve patient prioritization, streamline clinical operations, and ultimately contribute to better quality of care.

## INTRODUCTION

The recent global pandemic, coupled with an aging population, has led to a significant increase in demand for emergency medical assistance worldwide. In 2021, member countries of the Organization for Economic Cooperation and Development (OECD) with available data reported an average of 27 emergency department (ED) visits per 100 people annually^1^. Examples include Canada with 36.6 visits, England with 43.1, Australia with 34.2, and the United States of America with 43 visits per 100 people^2^. In Türkiye, the rate is significantly elevated, approximately 5.56 times the average of OECD countries^3^. When demand exceeds the capacity of EDs, several challenges in patient care can emerge. These include prolonged waiting times for medical evaluation, insufficient physical infrastructure, overburdened healthcare personnel, and delays in both treatment initiation and patient discharge^4^. In response to these challenges, emergency care in EDs typically begins with a triage process, which enables the prioritization of patients based on the severity and urgency of their medical needs.

Most widely adopted emergency department triage systems use five-level classification models. The most recognized are the Emergency Severity Index (ESI), Manchester Triage System (MTS), Canadian Triage and Acuity Scale (CTAS), and Australian Triage Scale (ATS), which form the basis of many global triage models. While most systems follow five levels, China uses a four-level system, the Chinese Emergency Triage Scale (CETS), whereas Türkiye uses a three-level, color-coded system called the Türkiye Triage System (TTS), established by a directive of the Turkish Ministry of Health in 2009^5^. TTS classifies patients into three levels: Red, Yellow, and Green, with Red and Yellow further divided into two subcategories^6^. Red represents the most critical cases: Subcategory 1 requires immediate intervention for life-threatening conditions (e.g., cardiac arrest, major trauma), while Subcategory 2 includes high-risk cases needing treatment within 10 min (e.g., severe chest pain, acute neurological symptoms). Yellow covers serious but less urgent conditions: Subcategory 1 involves risk of limb loss or significant morbidity (e.g., severe hypertension, moderate bleeding), and Subcategory 2 includes prolonged yet non-critical issues such as minor trauma or gastrointestinal complaints. Green refers to stable patients with mild symptoms suitable for outpatient management.

Effective triage implementation ensures appropriate patient prioritization while optimizing ED resource allocation^7^. Reliable triage systems should be based on validated scoring methods^8^; however, variations in data collection, cultural context, and individual or environmental factors may introduce assessment errors^9^. Integrating artificial intelligence (AI) can mitigate these issues by enhancing the precision, consistency, and overall effectiveness of traditional triage systems^10^. Machine learning (ML) identifies actionable patterns within complex datasets through computational algorithms. For instance, a Gradient Boosted Decision Tree model implemented with the CatBoost library was developed to support ED triage officers in identifying potentially life-threatening mis-triage cases based on CETS^11^. This approach significantly reduced mis-triage among critically ill patients and outperformed logistic regression (LR).

Several ML models, Support Vector Machine (SVM), k-Nearest Neighbors (KNN), Decision Tree (DT), and Random Forest (RF), were evaluated for triage in the TTS^12^, with RF achieving the best performance. Beyond outcome prediction, an XGBoost model was developed to recommend critical clinical interventions, improving timely decision-making^13^. An XGBoost-based model optimized with Hyperopt replicated clinicians’ decisions, reducing over-triage and under-triage while improving efficiency^14^. Another study compared multiple ML algorithms for predicting discharge, hospitalization, ICU admission, and mortality, using oversampling and genetic algorithms for optimization; RF emerged as the top model^15^. Additionally, six ML models integrating structured and unstructured triage data via NLP were evaluated against clinicians’ predictions, accurately forecasting ICU admission, mortality, and hospitalization, demonstrating their value in patient prioritization^16^.

This study aims to compare the performance of several supervised ML algorithms for three-class ED triage prediction within the Turkish triage system using routinely available triage variables. By evaluating these models on a retrospective, single-center dataset, we seek to assess their predictive performance and identify methodological considerations for future externally validated triage decision-support studies.

## METHODS

### Dataset

This study used anonymized patient data collected from the ED of Kayseri City Hospital. Ethical approval for data collection was obtained from the Non-Interventional Clinical Research Ethics Committee. The data includes structured triage information for adult patients (aged 18 and above), including demographic characteristics (e.g., age, gender), vital signs, and clinical complaints. Only patients with complete triage data and approved urgency classifications were included in the analysis. The dataset comprises 510 patients equally represented in three triage categories: red, yellow, and green. The features were selected based on their routine availability during ED triage and relevance to urgency classification. The dataset includes structured information on patient demographics, vital signs, and chief complaints recorded at triage. Demographic variables comprise age and gender. Vital signs include systolic and diastolic blood pressure, heart rate, respiratory rate, body temperature, and oxygen saturation. Chief complaints represent the primary condition reported at admission, with a total of 58 distinct complaints recorded in the dataset.

### Data preparation

We applied several preprocessing steps to the dataset to ensure machine readability and consistency. Initially, categorical values such as gender and urgency labels were encoded into numerical representations using label encoding, allowing for direct processing by algorithms. To ensure comparability between variables, the six vital sign features were normalized between 0 and 1. The preprocessed data was exported in the attribute-relationship file format, which allows for seamless integration with the WEKA ML environment.

### Machine learning approaches

This study applied a supervised ML approach to train eight models on the ED triage dataset: BayesNet, DT, KNN, LR, Multilayer Perceptron (MLP), RF, SVM, and Majority Voting (MV). We employed model evaluation using WEKA. Given the relatively small size of the dataset, extensive hyperparameter search was avoided; instead, limited tuning was performed for the main tunable classifiers using WEKA’s CVParameterSelection within the 10-fold cross-validation framework. BayesNet was kept with the standard WEKA configuration, and MV was reconstructed using optimized base classifiers. Table 1 summarizes the tuning strategy. Model performances were evaluated using accuracy, precision, recall, F1-score, specificity, and AUROC, and 95%CI for accuracy were calculated using the Wilson method.

**Table 1:** Hyperparameters used for the machine learning models.

Models	Tuned parameter(s)	Search range	Selected value
BayesNet	Not tuned	Standard configuration	Default
DT	Confidence factor	0.1–0.5	0.1
KNN	Number of neighbors	1–15	1
LR	Ridge parameter	1.0E-8–1.0E-4	1.0E-8
MLP	Learning rate	0.1–0.5	0.1
RF	Number of trees	50–300	50
SVM	Complexity parameter	0.1–10	2.575
MV	Not independently tuned	Optimized base classifiers	Majority voting

DT: Decision Tree; KNN: k-Nearest Neighbors; LR: logistic regression; MLP: Multilayer Perceptron; RF: Random Forest; SVM: Support Vector Machine; MV: Majority Voting.

### Implementation details

We performed data analysis and visualization in Python and classification experiments in WEKA due to its robust collection of ML algorithms and filters. Specifically, we applied the Partition Membership filter to transform the data into a propositionalized representation, as in^17^. This filter partitions the instance space using a partition generator based on DT models such as J48, RF, or REPTree and encodes membership values as new attributes. The resulting transformed instances are combined with the original class labels to form a sparse representation suitable for classification. This transformation allows traditional classifiers to be used on the data while preserving the essential structural information obtained from the partitioning process.

## RESULTS

In this section, we first explore the correlations between the features to understand their mutual relationships and then present the comparative performance of the ML models on the triage classification task.

Figure 1 presents the feature correlation matrix for the vital signs and TTS class. A positive correlation exists between diastolic and systolic blood pressure (r=0.69), aligning with physiological expectations. Heart rate shows a moderate positive correlation with respiratory rate (r=0.40), while oxygen saturation shows a moderate negative correlation with respiratory rate (r=-0.48). Correlations between individual vital signs and TTS class are generally weak to moderate, with the highest association observed for oxygen saturation (r=0.36), followed by respiratory rate (r=-0.20). The positive association between oxygen saturation and TTS suggests that higher saturation values are linked to less urgent triage categories, whereas the negative association of respiratory rate with TTS indicates higher respiratory rates in more urgent cases. Overall, no single vital sign strongly predicts triage class independently.

**Figure 1:**
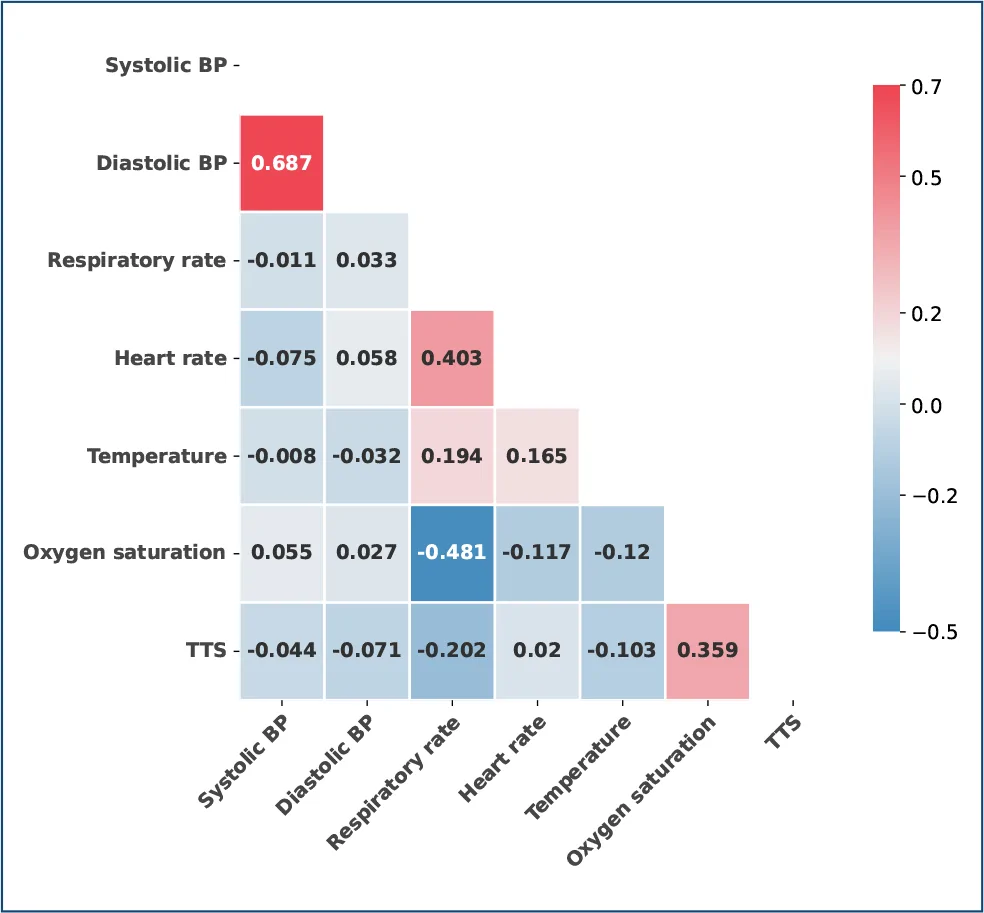
Correlation matrix of the features and Türkiye Triage System class.

We evaluated the performance of several ML algorithms to classify patients into urgency levels. All models were evaluated using a 10-fold cross-validation strategy to obtain an internal estimate of predictive performance. In each iteration, nine folds were used for training, and the remaining fold was used for testing. This process was repeated until each fold served once as the test set, and the final performance scores were averaged across all folds.

The results in Table 2 show that LR and RF yield the highest overall classification performance in most metrics. LR achieves the highest accuracy (0.902; 95%CI 0.873–0.925), precision (0.902), and specificity (0.951), while RF closely follows and achieves the highest AUROC. KNN, SVM, and MV also demonstrate competitive performance, with accuracy values close to 0.89. However, overlapping CI indicates small numerical differences among the best-performing models. In contrast, BayesNet underperforms across all metrics, with an accuracy of only 0.578 and an F1-score of 0.539, suggesting limited discriminative capacity on this dataset.

**Table 2:** Classification metrics comparison.

Methods	Accuracy (95%CI)	Precision	Recall	F1-score	Specificity	AUROC
BayesNet	0.578 (0.535–0.621)	0.621	0.578	0.539	0.789	0.786
DT	0.845 (0.811–0.874)	0.845	0.845	0.845	0.923	0.909
KNN	0.894 (0.864–0.918)	0.895	0.894	0.894	0.947	0.935
LR	0.902 (0.873–0.925)	0.902	0.902	0.902	0.951	0.931
MLP	0.880 (0.849–0.906)	0.880	0.880	0.880	0.940	0.935
RF	0.898 (0.869–0.921)	0.898	0.898	0.897	0.949	0.945
SVM	0.890 (0.860–0.914)	0.890	0.890	0.890	0.945	0.929
MV	0.892 (0.862–0.916)	0.892	0.892	0.892	0.946	0.919

DT: Decision Tree; KNN: k-Nearest Neighbors; LR: logistic regression; MLP: Multilayer Perceptron; RF: Random Forest; SVM: Support Vector Machine; MV: Majority Voting; AUROC: area under the receiver operating characteristic curve.

Figure 2 presents the confusion matrices, offering a detailed view of class-wise performance and highlighting prediction trends across urgency levels. LR, RF, SVM, and the MV ensemble demonstrate the most consistent and accurate results, with high true positive rates across all three TTS classes. In contrast, BayesNet performs substantially worse, frequently misclassifying TTS-2 and TTS-3, indicating limited ability to capture class overlap. DT and KNN show moderate performance. DT achieves reasonable accuracy, particularly for TTS-3, but often misclassifies TTS-1 as TTS-2, while KNN occasionally confuses TTS-1 and TTS-2, suggesting insufficient separation in local feature neighborhoods. MLP maintains a good balance across classes, though minor confusion between TTS-1 and TTS-2 persists, likely due to overlapping feature distributions.

**Figure 2:**
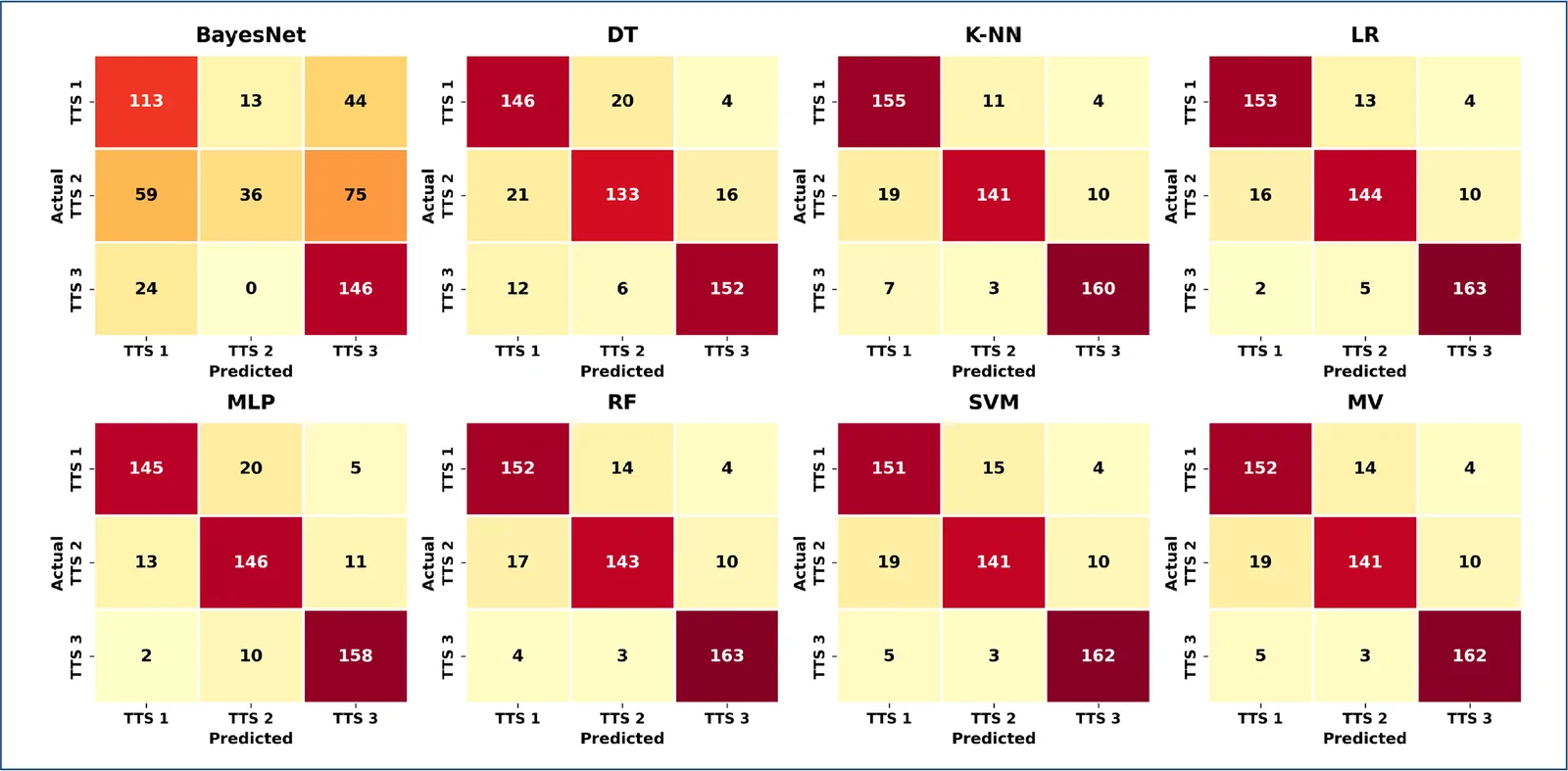
Confusion matrices obtained from a 10-fold cross-validation.

The dataset is class-balanced, with 170 instances per urgency level. Among all classifiers, TTS-3 is the most accurately detected class: LR, RF, SVM, and MV correctly classify 162 of 170 TTS-3 cases. In contrast, TTS-2 is the most challenging class, with BayesNet misclassifying nearly half of its instances, often as TTS-3. Performance on TTS-1 is generally stable, though DT and MLP occasionally confuse it with TTS-2.

## DISCUSSION AND CONCLUSION

This study evaluated several ML algorithms for three-class ED triage prediction using a balanced single-center dataset. LR achieved the highest numerical accuracy, while RF showed the highest AUROC. However, overlapping Wilson CIs indicate that differences among the best-performing models should be interpreted with caution. Additionally, correlation analysis showed that routinely measured vital signs, particularly oxygen saturation, contained relevant information for TTS classification; however, no single variable could independently explain triage severity. These findings suggest that machine learning–based models can support triage decision-making processes but should be considered as complementary tools rather than replacements for clinical judgment.

Several limitations must be considered. The relatively small sample size, single-center retrospective design, and lack of external validation may restrict generalizability. While the study aimed to compare ML algorithms within a controlled and balanced dataset, further validation using larger, prospective, and multi-institutional cohorts is necessary to confirm robustness, fairness, and clinical applicability in real-world emergency care.

## Data Availability

The datasets generated and/or analyzed during the current study are available from the corresponding author upon reasonable request.
